# Phosphoethanolamine: a translational journey from biological process and physiopathological effects to therapeutical innovation - a mini-review

**DOI:** 10.3389/fphar.2026.1790807

**Published:** 2026-04-23

**Authors:** Guilherme Ayres Rossini, Marcia Koike, Denise F. Barbeiro, Hermes Barbeiro, Daniel Rabelo, Francisco Garcia Soriano, Marcel Cerqueira Cesar Machado, Durvanei Augusto Maria

**Affiliations:** 1 Medical Investigation Laboratory 51 (LIM51) - Clinics Hospital Faculty of Medicine, University of São Paulo (HC-FMUSP), São Paulo, Brazil; 2 Faculty of Medicine, University of São Paulo, São Paulo, Brazil; 3 Development and Innovation Laboratory, Butantan Institute, São Paulo, Brazil

**Keywords:** 2-aminoethyl dihydrogen phosphate, ETNK1, ischemia reperfusion (I/R) injury, Pcyt2, phosphoethanolamine, phospholipid metabolism, succinate dehydrogenase (complex II)

## Abstract

Phosphoethanolamine (pETN), an endogenous metabolite in the Kennedy pathway for membrane phospholipid synthesis, can be chemically synthesized and exemplifies translational pharmacology through its journey from basic biochemistry to clinical application. Initially recognized for phosphatidylethanolamine biosynthesis via PCYT2-mediated conversion, pETN gained clinical attention following reports of Brazilian cancer patients with pETN-containing products. Subsequent pharmacological studies revealed favorable safety profiles in preclinical and clinical settings, with oral bioavailability of 6%–7%. Mechanistic investigations identified pETN as a competitive inhibitor of succinate dehydrogenase (complex II and Krebs cycle), providing molecular rationale for its mitochondrial effects. This discovery opened new therapeutic avenues beyond oncology, particularly in ischemia-reperfusion injury, where pETN’s succinate dehydrogenase inhibitory activity could mitigate pathological succinate accumulation and subsequent oxidative damage during reperfusion. The translational trajectory of pETN, from endogenous metabolism through clinical observation to mechanistic understanding, exemplifies the bidirectional nature of modern pharmacological research and positions this naturally occurring compound as a promising therapeutic candidate for mitochondrial dysfunction-related conditions.

## Introduction

Around 2015, Brazil was involved in a controversy regarding health care judicialization in which a considerable number of cancer patients consuming a phosphoethanolamine-based product sought legal action to gain access to the compound because they felt much better after consuming (Leford, 2015). Currently, in the United States of America (United States), phosphoethanolamine-based products are successfully sold as a dietary supplement, with one of the products with more than 70 reviews in Amazon’s online store. Interestingly, positive comments about product consumption are observed in reviews, just like the Brazilians felt better and went to court to acquire access to the product.

Phosphoethanolamine (pETN) is a bifunctional organic compound formed by a phosphate group and an amino group, connected by an ethyl chain (Table 1). In biological systems, the compound is known to be part of the polar region of membrane phospholipids. Data from The Human Metabolome Database platform (Wishart et al., 2018), whose pETN ID’s is HMDB0000224, indicate that many food sources contain pETN (HMDB, 2025). It is possible to detect the presence of pETN in different human body fluids such as urine (Cusworth, 1958), feces (Goedert et al., 2014), blood (Saxena et al., 2017), saliva (Rose and Kerr, 1958), and cerebrospinal fluid (Seki et al., 2005), indicating a role in important biological processes beyond membrane structure. It also can be found in the placenta (Tian et al., 2006) and also in the breast milk (Twelves et al., 1994), suggesting a role in fetal development and infant development, especially liver development (Guan et al., 2020). In addition to the pETN endogenous pathophysiological whole, it can be chemically synthesized and exogenously administered. In this work, we propose a mini-review not only on the aspects of endogenous, but also on exogenous synthetic pETN and its translational aspects.

**TABLE 1:** Phosphoethanolamine’s (pETN) chemical characteristics and physical properties.

Chemical characteristics	
Chemical formula	C_2_H_8_NO_4_P
Molecular weight	141.063 Da (Da)
IUPAC name	(2-aminoethoxy)phosphonic acid
CAS registry number	1,071-23-4
pH < 1: Act as diprotic acid, with phosphate group fully protonated, the amino group remains protonated	
pH 1 - 5: Exist as neutral zwitterion, predominant form in aqueous solution and solid state	
pH 6 - 11: Behaves as anion, phosphate group loses its second proton resulting in a net negative charge on the compound	
pH > 11: Amino group deprotonates, compound remains anionic, but with a neutral amino group	
Physical properties	
Melting point	241 - 243 °C
Water solubility	72 mg/mL (aprox. 0.51 mol/L)

### Endogenous role and kennedy pathway

How and which metabolic reactions is pETN involved? It can act as both substrate and product of different enzymatic reactions in the body. Sphingosine-phosphate lyase-1 (SGPL1) is capable of cleaving the bioactive lipid molecule sphingosine-1-phosphate into pETN, which can be directed toward other metabolic pathways. Researchers suggest that SGPL1 may be involved in the pathogenesis of neurodegenerative diseases due to its involvement with autophagy in neurons (Mitroi et al., 2017). At the same time, pETN can be degraded by phosphatases enzymes such as phosphoethanolamine/phosphocholine phosphatase (PHOSPHO1), which is a phosphatase that hydrolyzes pETN into ethanolamine (ETN) and inorganic phosphate which will form apatite crystal in bone mineralization processes (Dillon et al., 2019). PHOSPHO1 is a targetable enzyme, lansoprazole acts as a noncompetitive inhibitor of PHOSPHO1 activity (Dillon et al., 2019). Another enzymatic reaction that pETN is involved is with ethanolamine-phosphate phospho-lyase (ETNPPL or AGXT2L1), which is a phospholyase capable of breaking down pETN into inorganic phosphate, ammonia, and acetaldehyde (Veiga-da-Cunha et al., 2012).

**FIGURE 1:**
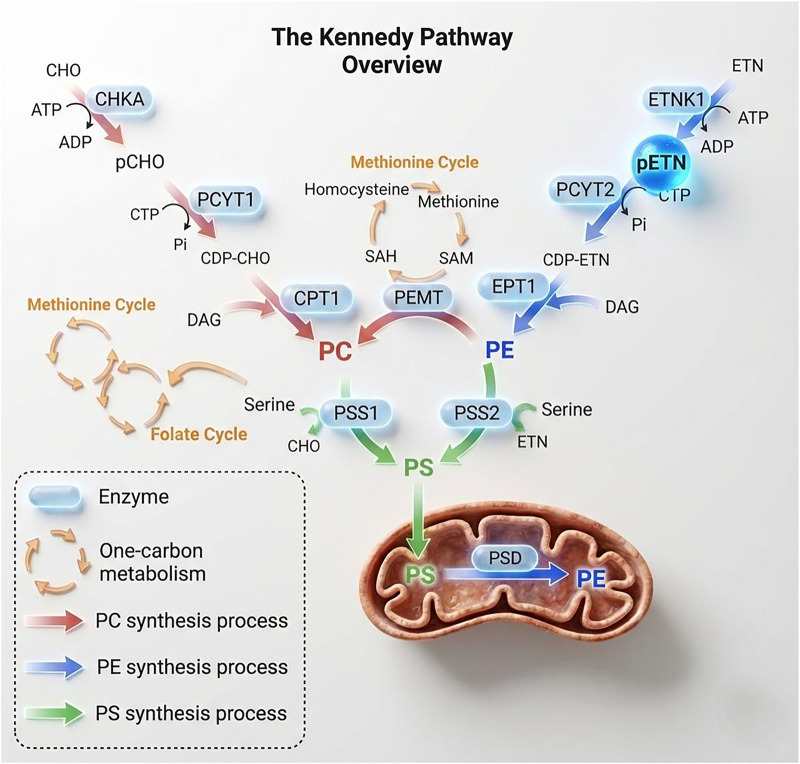
The canonical Kennedy Pathway overview represented by the two branches of the main phospholipid biosynthesis. Image adapted from the original work of Rossini and Maria (2023) and enhanced using AI-assisted editing for improved visualization.

The canonical pathway involving pETN is the Kennedy pathway (Kennedy and Weiss, 1956) for membrane phospholipid synthesis, a well-established biochemical and metabolic process (Gibellini and Smith, 2010; Dowhan et al., 2021). In this pathway, which is represented in Figure 1, ethanolamine kinase-1 (ETNK1) and ethanolamine kinase-2 (ETNK2), choline kinase-A (CHKA) and choline kinase-B (CHKB) are four enzymes responsible for the conversion of ETN to pETN. All utilize ATP for substrate ETN phosphorylation, dysfunction of those enzymes are associated with different pathologies. The CHKA enzyme is involved in the physiopathology of some neurodevelopmental disorders, severe epilepsy, and microcephaly (Klockner et al., 2022). CHKB is involved in the physiopathology of a specific muscular disorder called CHKB-related muscular dystrophy, where cells with low activity of this enzyme have lower phosphatidylcholine (PC) levels in mitochondrial membranes, rendering them dysfunctional for beta-oxidation (Chan and Nishino, 2023). ETNK2 has been associated with placental thrombosis in animal models, possibly due to the relationship between phosphatidylethanolamine (PE) and hemostasis via protein C (Tian et al., 2006). In addition to ETNK1 (Fontana et al., 2020), all four enzymes (ETNK1/2, CHKA/B) responsible for ETN phosphorylation to pETN are associated with tumors (Hu et al., 2016; Zhang et al., 2016; Huang et al., 2022; Chu et al., 2023).

In the canonical pathway (Figure 1), after the conversion of ETN into pETN, pETN acts as a substrate for the enzyme CTP:phosphoethanolamine cytidylyltransferase-2 (PCYT2) - a highly specific for pETN, which converts pETN to CDP-ETN. The PCYT2 enzyme transfers a cytidine monophosphate (CMP) group to pETN, activating it for the next synthesis step. Finally, 1,2-diacylglycerol ethanolamine phosphotransferase (EPT1 or SELENOI) condenses CDP-ETN with diacylglycerol (DAG) to form PE (Pavlovic and Bakovic, 2013). PE can be converted to PC, which stands out as the most abundant membrane phospholipid in mammals, by the enzyme phosphatydylmetyltransferase enzyme (PEMT), which transfers methyl groups from S-adenosylmethionine (SAM) (Ye et al., 2017), this suggests a connection between the phospholipid metabolism with the one-carbon metabolism (Ye et al., 2017), the phosphatidylserine metabolism is also connected to one-carbon metabolism through serine (Yang and Vousden, 2016). The conversion of PE to PC is important for regulating the proportion of phospholipids in cellular membranes. Finally, PE also regulates critical cellular processes such as membrane fusion, protein anchoring, and autophagy (Rockenfeller et al., 2015). Overall, this pathway is involved in different physiological and pathological processes (Patel and Witt, 2017).

One of the main enzymes involved in the PE branch of the canonical Kennedy pathway (Figure 1) is PCYT2, controlled assays in mice showed that total knockout (−/−) of the PCYT2 enzyme is incompatible with animal life. Embryos died around 8.5 days of life. These data suggest that the canonical Kennedy pathway is an important and essential pathway for the survival of living organisms (Fullerton et al., 2007). Similarly, other assays using human hepatic organoids (HOs) and hepatoblasts (HBs), the authors observed a coordinate upregulation of all three enzymes (ETNK1, PCYT2, EPT1) in HBs, with significant increases in their mRNA levels (75-, 58-, and 41-fold, respectively) compared to control, crucially, the abundance of pETN itself was dramatically increased (>170-fold) in HBs compared to control, this indicate that the kennedy canonical pathway, specially PCYT2 and pETN is essential for liver development (Guan et al., 2020). Partial knockout studies (PCYT2^+/−^) associated the pathway with the physiopathology of non-alcoholic steatohepatitis, revealing a connection between phospholipid metabolism and insulin resistance, obesity, hepatic steatosis, and dyslipidemia (Grapentine et al., 2022). The mechanism is related to decreased DAG consumption and consequent accumulation, the cell increases fatty acid and triacylglycerol (TG) synthesis (Grapentine et al., 2022). The studies and findings about PCYT2 and the Kennedy canonical pathway reveals a whole new layer of therapeutic possibilities by metabolic modulation of the pathway, for example: PCYT2 is a targetable enzyme (Gohil et al., 2013).

Meclizine, an FDA-approved antiemetic drug, directly inhibits PCYT2 activity in a dose-dependent, non-competitive manner. This inhibition leads to a significant accumulation of the substrate pETN (Gohil et al., 2013). Experimental evidence supports that HepG2 cells treated with 40 µM meclizine showed a 3.7-fold significant increase in cellular pETN after 24 h, while ethanolamine levels remained unchanged this elevated pETN then acts as the direct mitochondrial perturbing agent: inhibition of mitochondrial respiration and membrane potential alteration which generates toxicity (Guan et al., 2020). While meclizine alone demonstrated potent cytotoxicity against developing liver cells, its effect on established HCC cell lines in standard glucose media was limited, however, when combined with PFK158 (a third-generation inhibitor of PFKFB3, an enzyme that stimulates glycolysis), a significant synergistic effect on cell growth was observed in both HepG2 and HuH7 hepatoma cell lines. The study didn't stop at *in vitro* results, it provided compelling *in vivo* evidence using a human HCC xenotransplantation model in mice: the combination of meclizine and PFK158 substantially reduced tumor burden in the treated mice with no toxicity to the animals (Guan et al., 2020). The efficacy was also demonstrated in primary acute myeloid leukemia (AML) blasts *in vitro*, showing a synergistic effect of meclizine and PFK158 in eliminating these cells, suggesting that Kennedy canonical pathway manipulation could be a used synergistically, with others drugs, in a broader range of cancers, not just HCC, emerging as a crucial target for cancer chemotherapy (Guan et al., 2020).

### Synthetic approaches and physicochemical properties

Due to its structure, pETN exhibits unique properties (Table 1), including the ability to form zwitterions and interact with metallic ions and organic compounds. In fact, it exists predominantly as a zwitterion in solid state, as well as in aqueous solutions in the pH range between 1 and 5. In this context, the amino group is protonated (NH3+), while one of the phosphate groups is deprotonated (OPO3H-), resulting in a neutral compound with opposite charges (Myller et al., 2013). The phosphate group undergoes two deprotonations at pH 1.0 and 5.9, while the amino group deprotonates at pH 11 (Table 1). These characteristics imply an “adaptive” behavior in response to different pHs. At the same time, the phosphate group of pETN is capable of binding to various metallic oxides, such as TiO2 and iron oxides. Similarly, the amino group can bind to complex metallic ions and organometallic compounds. No less important, pETN can interact with organic biomolecules via hydrogen bonds, a relevant property in biological systems (Myller et al., 2013).

Edgard Laurence Outhouse was the first to describe a methodology for pETN synthesis with an 80% yield (Outhouse, 1936; Outhouse, 1937). In 1951, Italian scientists Giorgio Ferrari and Emiliano Ferrari patented an alternative synthetic route for pETN (Ferrari and Ferrari, 1951). In the same decade, Cherbuliez et al. (1959) proposed another synthetic route based on Outhouse’s methodology. In the same decade, researchers successfully separated phosphoethanolamine from cilatin (Neuzil et al., 1969). German physician Hans A. Nieper requested chemist Dr. Franz Kohler to synthesize the pETN salt. The chemical synthesis of pETN performed by Franz Kohler contained calcium and therefore became known as Calcium-EAP (Supplementary Figure S1A). Dr. Nieper initiated clinical studies of Calcium-EAP in multiple sclerosis in 1964, and by 1967, German federal legislation permitted the administration of Calcium-EAP for multiple sclerosis as a therapeutic option (Nieper, 1989). Nieper also observed that among more than 2,800 patients under Calcium-EAP successfully treated for multiple sclerosis, proportionally few developed cancer.

In Brazil, chemist Gilberto Orivaldo Chierice (1944–2019) proposed a novel synthetic route for pETN (Ribeiro Filho and Chierice, 1998). In Chierice’s synthetic route, monoethanolamine (ETN) and phosphoric acid are utilized under specific conditions for the chemical reactions to result in the formation of crystallized pETN salt (Supplementary Figure S2A) (Ribeiro Filho and Chierice, 1998; Al-Asfour, 2008). Chierice died at the age of 75 in June 2019. After his death, his group created one of the dietary supplements sold on Amazon’s online store, which contains pETN associated with calcium, zinc, and magnesium in its composition (Phosphopure, 2025). Studies conducted at the Unicamp laboratory that analysed Chierice pETN-USP and they found a purity of 32.2% of pETN, with other components such as ETN (18.2%) and phosphobisethanolamine (3.9%), inorganic phosphates (34.9%), and water (7.2%). This indicates that pETN-USP is found combined with calcium phosphate, magnesium, zinc, iron, and other metallic ions (Barreiro and Dias, 2016). When compared with pETN-Sigma Aldrich standard purity, there are some subtle differences in pharmacological profile (Supplementary Table S1A).

### Pharmacological profile and safety

The zwitterionic physicochemical characteristic of pETN (Table 1) (Myller et al., 2013) may have been responsible for the significant challenges encountered by the team at the Brazilian Center for Innovation and Preclinical Trials in Florianópolis (CIEnP) (Brasil, 2015). The researchers needed to develop a methodology for detecting pETN in plasma, which was validated with a restriction: that samples were processed and analyzed immediately after collection (Brasil, 2015). CIEnP made important pharmacological studies comparing pETN made by USP-São Carlos and pETN made by Sigma Aldrich (standard) (Supplementary Table S1A). Interestingly, even with similar absolute bioavailability (6.3% USP-São Carlos and 7% Sigma-Aldrich), they found significant differences between them in different aspects such as at half-life and time to reach the maximus concentration (Tmax) when orally administered (Supplementary Table S1A).

Beyond information listed in the Supplementary Table S1B, the CIEnP’s report stated that when intravenously administered, pETN presented an extremely short half-life: less than 5 min. The plasma concentration of the substance returned to endogenous levels in only 20 min. The oral half-life >60x that of intravenous indicates slower elimination after oral administration, possibly due to prolonged absorption or first-pass metabolism (Brasil, 2015). This behavior is characteristic of a “Flip-Flop” pharmacokinetic profile, where the absorption rate is slower than the elimination rate (Brasil, 2015).

In another study about pharmacological characteristics, synthetic ETN and pETN, satisfied Lipinski’s and Veber’s rules for molecular properties and drugability, indicating its suitability for oral administration in humans. However, while ETN demonstrated excellent stability in simulated gastric and intestinal fluids (SGF and SIF) for extended periods, pETN showed approximately 35% degradation in SIF after 2 h. The volume of distribution (VD) is high (4.82 L/kg) indicating that pETN distributes widely to tissues beyond blood volume. Excretion is represented through clearance determination, pETN showed high clearance (101.92 mL/min/kg), which indicates efficient elimination (Saxena et al., 2017). No significant drug interactions mediated by major cytochrome P450 (CYP) enzymes were observed (Saxena et al., 2017), however, there is no data about drug-drug interactions. Importantly, oral administration of pETN resulted in similar exposure for both pETN and ETN, suggesting that pETN can be converted to ETN *in vivo* by alkaline phosphatase enzymes present in the intestine and liver. This is an interesting finding in contrast to ETN’s route inside the cell, where it is converted to pETN at the canonical Kennedy Pathway (Saxena et al., 2017).

Overall, animal pre-clinical data indicates that pETN is safe (Supplementary Table S1B) (Bento et al., 2016; De Andrade et al., 2016a; De Andrade et al., 2016b; Moreira et al., 2019; Freitas et al., 2016; Brasil. Ministério da Ciência e Tecnologia e Inovação. Centro de Inovação e Ensaios Pré-Clínicos, 2016). In humans, Prof. Dr. Paulo Marcelo Gehm Hoff led the first controlled studies of orally administered pETN salt. The São Paulo State Government sponsored the study at Cancer Institute of São Paulo State (ICESP, 2019). The salt was considered safe and went to the second phase (ICESP, 2019). Together with phase two adverse events data, it can be concluded that oral administration of the salt was considered safe in humans, at the tested dosages (1.5 g/day), and no adverse effects were attributed to the use of pETN.

Via intravenous route, a work sought to evaluate acute and repeated-dose toxicity of pETN in Balb-c mice, overall, the data indicate safety at all repeated doses evaluated (50, 100, and 250 mg/kg) with only some minor transient alterations. In the evaluation of acute toxicity at a single dose, it also showed safety at doses 50, 100, 250 mg/kg and signs of toxicity at doses 500 and 1,000 mg/kg. The absence of significant toxicity at high doses is a positive finding that suggests pETN possesses a favorable safety profile (Araujo and Maria, 2018). In another study, this time a phase I trial in domestic dogs with intravenous administration of pETN, enrolled a total of 12 animals divided into 4 groups receiving different doses ranging from 30 mg/kg to 150 mg/kg administered intravenously weekly for up to 8 weeks. A transient change in respiratory rate was observed in some animals at the time of infusion. Despite this, the compound was considered safe up to the maximum dose tested (150 mg/kg) and did not influence biliary parameters or induce hepatotoxicity, nor did it alter renal function (urea and creatinine). Total protein and glycemic levels showed no significant difference, and electrolyte levels of potassium, sodium, and phosphorus remained constant, with only ionized calcium showing a significant increase in plasma levels compared to the control group (Cunha, 2019).

In dogs with high-grade mast cell tumors, weekly intravenous administration of pETN at doses of 70 mg/kg also proved to be safe and well tolerated by the animals (Januario et al., 2022). In another study involving dogs with soft tissue sarcoma, intravenous administration of pETN at doses of 70 mg/kg once weekly for 4 weeks revealed significant reduction in tumor temperature detected through infrared cameras (de Castro et al., 2022). This implies that strongly exists a metabolic change induced by pETN, that is not reflected in anatomical studies (RECIST). The reduction of tumor temperature after pETN intravenous administration observed, suggests tumor metabolic alteration induced by pETN (de Castro et al., 2022). Despite the safety data described, it is important to highlight the lack of long-term safety data, including the long-term biological effect and the potential risk of metabolic disturbance.

### Mechanisms of action and therapeutic potential

To date, there is not enough evidence for any recommendation about pETN efficacy in curing any human cancer. For skin cancer (melanoma), at ICESP study, one patient from the melanoma group presented significant tumor response according to RECIST v1.1 criteria (ICESP, 2019). Interestingly, pre-clinical study with mice grafted with melanoma showed a 64% reduction in tumor mass at doses of 1,000 mg/kg administered orally (Moraes Filho et al., 2016), others cell models and animal models also indicate pETN anti-tumor action against melanoma (Ferreira et al., 2011; 2012b). On the other hand, an interesting metabolic study suggests that pETN may have a cytoprotective effect on cancer cells (Osawa et al., 2019). Actually, overall data is highly suggestive of pETN cytotoxic effect is concentrations dependent. In addition, the cytotoxic concentration for tumor cells mitochondrias does not affect normal cells mitochondrias (Ferreira et al., 2011).

The canonical Kennedy’s pathway is known as a targetable anti-cancer pathway, as shown in the meclizine combined therapy study (Guan et al., 2020). However, the cytotoxicity meclizine’s mechanism of action involves pETN accumulation and mitochondrial respiration inhibition (Gohil et al., 2013; Guan et al., 2020). In fact, evidence from different research groups indicates that pETN acts as an inhibitor of mitochondrial respiration, manifesting as reduced oxygen consumption rates and altered mitochondrial membrane potential (Modica-Napolitano and Renshaw, 2004; Gohil et al., 2013; Guan et al., 2020). The increase in intracellular pETN levels (approximately 40-fold in prostate cancer model cells), leads to downregulation of HIF-1α, which leads to significant reduction in intracellular glucose and glutamine levels, in addition to downregulation of glycolytic enzymes and glutamine metabolism enzymes, exacerbating the metabolic crisis (Saxena et al., 2017). Studies from the Butantan Institute showed similar anti-tumoral effect: when pETN concentration increases at a certain level, the metabolic arrest mainly manifested as programmed cell death with involvement of mitochondria apoptotic pathway (Luna et al., 2018; Ferreira et al., 2012a; Ferreira et al., 2012b; Ferreira et al., 2013a; Ferreira et al., 2013b; Ferreira et al., 2013c).

**FIGURE 2:**
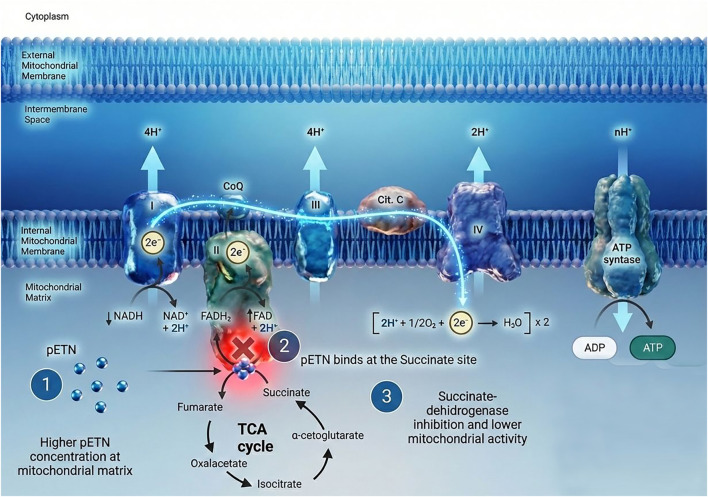
(1) Higher levels of pETN in the mitochondrial matrix induced by exogenous synthetic pETN administration; (2) Mitochondrial Electron Transport Chain’s Complex II (Succinate dehydrogenase or SDH) activity being inhibited by phosphoethanolamine (pETN); (3) SDH inhibition leading indirect interference with other complexes and lower mitochondrial activity. Image adapted from the original work of Rossini and Maria (2023) and enhanced using AI-assisted editing for improved visualization.

In a study with ETNK1 mutations, that impair ETNK1’s enzymatic activity, leading to a significant decrease in the intracellular concentration of pETN. As a consequence of reducing pETN, it causes a significant increase in mitochondrial activity. The mitochondrial hyperactivation is not due to altered membrane structure, but rather a more direct metabolic effect of pETN deficiency, a 1 mM exogenous pETN treatment led to a complete restoration of normal mitochondrial membrane potential, effectively reversing the hyperactivation phenotype, confirming that pETN acts as a negative regulator of mitochondrial respiration (Fontana et al., 2020). The study also evaluated the precise molecular mechanism that pETN directly impairs mitochondrial function by investigating the activity of mitochondrial complexes I, II, III, and IV in the presence of increasing pETN concentrations, while pETN had no significant effect on complexes I, III, and IV, it revealed a profound, dose-dependent decrease in the redox activity of mitochondrial complex II. To understand how pETN inhibits complex II, competition assays were performed with pETN and varying concentrations of succinate, the endogenous substrate of succinate dehydrogenase (SDH). The results showed that while pETN alone significantly decreased complex II activity, subsequent supplementation with succinate was able to restore normal SDH activity. This strongly suggests that pETN acts as a competitive inhibitor of succinate for SDH activity (Figure 2) (Fontana et al., 2020).

In addition, while there is evidence of pETN inducing mitochondrial membrane potential alterations (Modica-Napolitano and Renshaw, 2004; Gohil et al., 2013; Guan et al., 2020), it is known that complex II does not directly contribute to mitochondrial membrane potential (Cecchini, 2003). This suggests that pETN does not interfere exclusively with the function of SDH and complex II, but other mitochondrial complexes also could be involved, such as complex I, III and IV. Overall, an explanation for this phenomena can be described by: first, pETN directly inhibits SDH and complex II (Fontana et al., 2020). Second, complex II is not directly involved with the proton pump, but it contributes to electron flux that allows complex III and IV pump protons (Cecchini, 2003). Third, when complex I is compromised such as hypoxic conditions, complex II becomes essential for preserving membrane potential and mitochondrial function, including the ability to sequester calcium (Hawkins et al., 2010). Therefore, pETN complex II inhibition results in indirect interference with other complexes leading to mitochondrial membrane potential depolarization (Figure 2).

Mitochondrial membrane potential is dynamic and this depolarization pattern induced by pETN was observed systematically by our group in differentiated cell lines (used as control) and tumor cell lines such as human leukemia cells (Conceição et al., 2021), human and murine melanoma cells (Chammas and Maria, 2023; Felthes and Maria, 2024), human squamous cell carcinoma (Carvalho and Maria, 2017), human and murine triple-negative breast cancer cells (Cabral and Maria, 2021), Ehrlich ascites tumor cells (Alves and Maria, 2022), human non-small lung cell carcinoma (Ferreira and Maria, 2013), murine renal carcinoma (Ferreira and Maria, 2013), human glioblastoma (Medina and Maria, 2023), murine hepatocarcinoma (Luna et al., 2018).

### Future perspectives and clinical translation

This novel mechanism, provides a clear molecular explanation for meclizine’s cytoprotective effects observed in models of ischemia-reperfusion injury and neurodegeneration, meclizine pretreatment conferred dose-dependent protection against cell death in simulated ischemia-reperfusion injury in adult rat cardiomyocytes (Gohil et al., 2010). The metabolic signature of ischaemia is the universal accumulation of succinate (Chouchani et al., 2014). Ischaemia-reperfusion or IR injury occurs when blood supply is interrupted and then restored. While reperfusion is necessary for tissue survival, it paradoxically initiates oxidative damage and cell death. During ischaemia, succinate levels dramatically increase (3–19-fold in various tissues) (Chouchani et al., 2014). This occurs because SDH, under anaerobic conditions, can operate in reverse, reducing fumarate to succinate. Fumarate, in turn, is supplied by pathways like the malate/aspartate shuttle and the purine nucleotide cycle. Upon reperfusion, oxygen becomes available again. The accumulated succinate is then rapidly re-oxidized by SDH. This re-oxidation event drives extensive electron charge into electron transport chain leading to reverse electron transport (RET) at mitochondrial complex I which leaks into the mitochondrial matrix and reacts with oxygen from reperfusion generating superoxide radical (ROS) (Chouchani et al., 2014). This RET mechanism leads to the oxidative damage characteristic of IR injury. Could pETN, identified as a natural SDH inhibitor (Fontana et al., 2020), be therapeutically beneficial in ischaemia-reperfusion injury?

Actually recent experiments in our laboratory suggest that pETN reduces the accumulation of succinate in ischemic liver therefore reducing the effects of ischemic reperfusion injury (unpublished), pETN might achieve a dampening effect on SDH activity during reperfusion, thereby reducing the burst of mitochondrial ROS and mitigating IR injury. This would be a novel application for pETN. The capacity of pETN to act as a competitive inhibitor of mitochondrial complex II (SDH) provides a direct mechanistic link to the subject of ischaemia-reperfusion injury. Both phenomena revolve around the precise control of SDH activity and its impact on mitochondrial ROS. On one hand, a scientific method data show what happens when SDH is overwhelmed by substrate during reperfusion (Chouchani et al., 2014), on the other hand, another scientific method data demonstrate the consequences when SDH loses its natural endogenous inhibitor (Fontana et al., 2020). Understanding this dual control of SDH activity is crucial for comprehending mitochondrial ROS generation in a wide array of pathological conditions. That could turn out to be potentially new opportunities for development of therapeutic strategies applications related to IR injury, such as organ transplantation (Martin et al., 2019). At the same time, the pETN application in IR injury could lead to adverse effects from the excessive SDH inhibition.

It is important to point out that synthetic pETN can be considered as a biotechnology, with interesting differences between the brazilian pETN-USP vs. pETN-Sigma Aldrich (Supplementary Table S1A). Another important difference is the cost of biotechnology where the pETN-USP, from Chierice`s synthesis route (Supplementary Figure S2) is much cheaper than pETN-Sigma Aldrich. This biotech has a huge potential in a multitude of pathology scenarios, considering the IR injury field and emergency department, it could help patients with different types of shock, including sepsis. In addition, it is possible to observe higher endogenous pETN liquor levels in patients with traumatic brain injury, considering that traumatic brain injury is a process that involves local IR injury (Seki et al., 2005), high pETN levels might be a protective cellular mechanism to dampen mitochondrial overactivity or mitigate ROS production by inhibiting SDH.

At this point, data is clear that delivery details matter when it comes to pETN, when it is administered by gastrointestinal tract such as oral or feeding tube its absorption is limited at the same time that is safe to use (Supplementary Table S1B). Lansoprazole, which has an inhibitory effect on phosphatase that hydrolyzes pETN (Dillon et al., 2019), could be combined as a strategic alternative to this oral limited bioavailability. At the same time, there is no data about pETN effect on gut microbiome modulation, future studies that involve pETN oral or feeding tube administration could focus on this system, if the intestinal bacteria can be selectively stimulated by pETN leading to host’s health improvement as consequence, pETN could also be considered a prebiotic compound (Davani-Davari et al., 2019). In addition, an improvement of the biotech with liposomal drug delivery system with pETN formulation expands the potential. Our group has focused on cationic liposomes, primarily utilizing Dioctadecyldimethylammonium Chloride (DODAC) (Carvalho and Maria, 2017; Bonfim Neto and Maria, 2018; Chammas and Maria, 2023; Luna et al., 2018; Silva and Maria, 2016) and Dioctadecyldimethylammonium Bromide (DODAB) (Gomes and Maria, 2024). The positive surface charge of these liposomes facilitates electrostatic interactions with negatively charged cellular components, enhancing drug encapsulation efficiency and potentially improving cellular uptake, especially in cancer cells (Luna et al., 2018). The development of these advanced formulations is underpinned by physicochemical characterization, this includes dynamic light scattering, zeta potential measurements and electron microscopy (Luna et al., 2018; Gomes and Maria, 2024). Despite these significant advancements, the field of liposomal formulation continues to present challenges and opportunities for optimization. Maintaining long-term stability, achieving controlled release kinetics, and potentially incorporating active targeting ligands, such as peptides and antibodies, for even greater specificity are ongoing areas of research specially in the precision oncology field (Su et al., 2026).

## Conclusion

This mini-review exemplifies the bidirectional nature of translational biotechnology, illustrating how pETN research has evolved through multiple scientific cycles in different countries. What began as basic chemical alchemy studies and clinical observation, drove the discovery of pETN’s novel mechanism as a succinate dehydrogenase inhibitor. This mechanistic understanding now opens new therapeutic avenues, particularly in IR injury, where pETN’s ability to competitively inhibit SDH could mitigate the pathological succinate accumulation that drives oxidative damage during reperfusion. The translational journey of pETN demonstrates how fundamental metabolic research, clinical observations, and mechanistic discoveries can synergistically advance therapeutic innovation, positioning this endogenous metabolite and its synthetic version as a promising candidate for diverse pathological conditions involving mitochondrial dysfunction.

## Supplementary material

SUPPLEMENTARY FIGURE S1 The key milestones of pETN synthesis route discovery. The image was made using figma platform and enhanced using AI-assisted editing for improved visualization.

SUPPLEMENTARY FIGURE S2 Chierice’s synthesis route for pETN crystals salt formation. Image adapted with figma platform from original Brazilian patent application BR-PI0800460-9 A2 and enhanced using AI-assisted editing for improved visualization.
