# Supplementary material for: Phosphoethanolamine: a translational journey from biological process and physiopathological effects to therapeutical innovation - a mini-review
Source: Front Pharmacol. 2026 Apr 23;17:1790807. doi: 10.3389/fphar.2026.1790807 (PMC13149127; doi:10.3389/fphar.2026.1790807)
Supplement: Supplementary file 1 [file Table1.docx]

**Supplementary Table-S1: A.** Pharmacological Properties comparing pETN-USP vs pETN-Sigma Aldrich (standard); **B.** Safety profile.

| 1. **Pharmacological Properties** | | | | | |
| --- | --- | --- | --- | --- | --- |
| **Absolute bioavailability (oral)** | | pETN USP: 6,7% | | pETN Sigma: 7% | |
| **Half Life (oral)** | | pETN USP: 24 hours | | pETN Sigma: 5 hours | |
| **Tmax (oral)** | | pETN USP: 5 to 360 minutes | | pETN Sigma: 5 to 120 minutes | |
| 1. **Safety profile** | | | | | |
| - **Mutagenicity (Ames test):** *S. typhimurium* strains, absence of DNA damage (Brasil, 2016); - **Genotoxicity (Micronucleus):** Swiss mice (500-2000 mg/kg oral) (Freitas, 2016); - **Bone marrow analysis:** no clastogenic/ aneugenic potential (Araujo, 2018). | | | | | |
| - **Cardiotoxicity (hERG):** HEK293 cells expressing Kv11.1, low risk of torsades de pointes (Moreira CG, 2019); - **Cardiovascular Safety:** wistar rats (10-100mg/kg intravenous), hemodynamic monitoring, no acute cardiovascular effects (De Andrade, 2016a); - **Neurotoxicity:** Wistar rats (30-300mg/kg oral), no neurological alterations, normal reflexes, safe for the central nervous system at tested doses (De Andrade, 2016b). | | | | | |
| - **Lethal Dose 50%:** wistar rats (2000 mg/kg oral, single dose), no mortality, no clinical signs of toxicity (Bento, 2016) - **Single Dose Toxicity:** Balb-c mice (50, 100, 250, 500 and 1000 mg/kg intravenous), no significant alterations at doses 50, 100, 250mg/kg, alterations in consciousness state, abdominal contortions, muscle contractions, tremors and convulsions at high doses (500 and 1000 mg/kg), significant mortality of 33% of animals receiving 1000 mg/kg (Araujo, 2018) - **Repeated Dose Toxicity:** wistar rats (1000 mg/kg/day oral for 7 days), no hematological, biochemical alterations, normal organ weights (Bento, 2016) - **Repeated Dose Toxicity:** balb-c mice (50, 100 and 250 mg/kg intravenous - 6 doses in 2 weeks), no mortality, no significant clinical and biochemical alterations, alterations in erythrocyte, leukocyte, platelet and reticulocyte levels, with dose- and time-dependent variations(Araujo, 2018) - **Repeated Dose Toxicity:** domestic dogs (4 groups 30 to 150mg/kg intravenous), no significant alterations, blood tests remained normal overall (Januario et al., 2022) (Cunha, 2019) - **Repeated Dose Toxicity:** Humans (1500mg/day oral once daily), no signs of toxicity, no significant alterations in hematological and biochemical parameters (ICESP, 2019) | | | | | |
