# Supplementary figures and images for: Phosphoethanolamine: a translational journey from biological process and physiopathological effects to therapeutical innovation - a mini-review
Source: Front Pharmacol. 2026 Apr 23;17:1790807. doi: 10.3389/fphar.2026.1790807 (PMC13149127; doi:10.3389/fphar.2026.1790807)

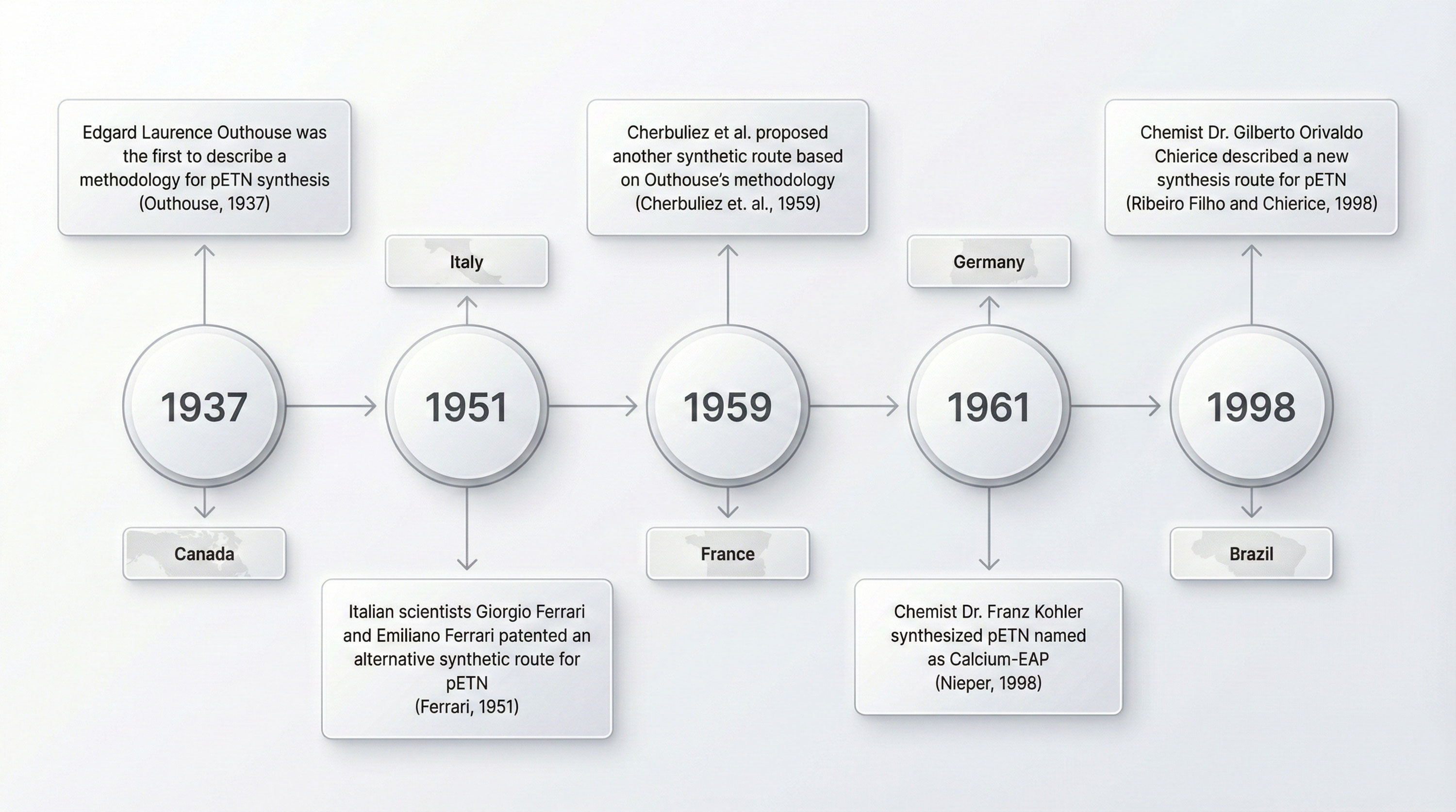

Supplement: Supplementary file 2 [file Image1.jpeg]

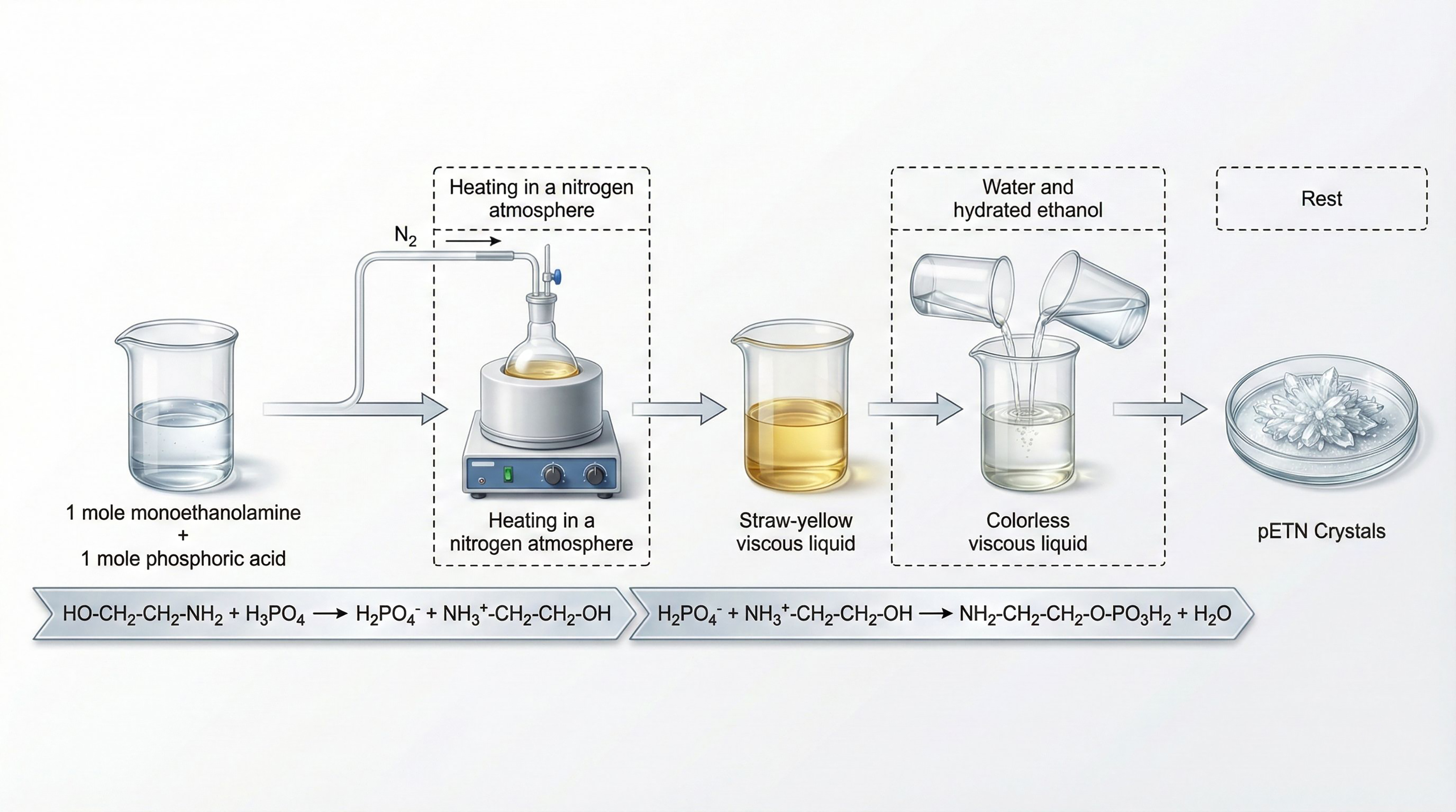

Supplement: Supplementary file 3 [file Image2.jpeg]
